# Immune checkpoint inhibitors as first line in advanced melanoma: Evaluating progression‐free survival based on reconstructed individual patient data

**DOI:** 10.1002/cam4.5067

**Published:** 2022-08-03

**Authors:** Andrea Ossato, Vera Damuzzo, Paolo Baldo, Daniele Mengato, Marco Chiumente, Andrea Messori

**Affiliations:** ^1^ Department of Pharmaceutical and Pharmacological Sciences University of Padova Padova Italy; ^2^ Centro di Riferimento Oncologico di Aviano IRCCS Aviano Italy; ^3^ Italian Society of Clinical Pharmacy and Therapeutics‐SIFaCT Milan Italy; ^4^ HTA Unit, Regione Toscana, Regional Health Service Florence Italy

**Keywords:** advanced melanoma, individual patient data, IPDfromKM method, ipilimumab, nivolumab, patient data reconstruction, pembrolizumab, progression‐free survival, relatlimab, survival meta‐analysis

## Abstract

**Background:**

In patients with advanced melanoma, immune‐checkpoint inhibitors (ICIs) represent the mainstay for first line treatment. Recently, relatlimab+nivolumab was proposed as a new combination therapy. This review was aimed at summarizing the current data of effectiveness for ICIs. Progression‐free survival (PFS) was the endpoint of our analysis.

**Methods:**

After a standard literature search, Phase II/III studies comparing different ICI regimens in previously untreated advanced melanoma patients were analyzed. Patient‐level data were reconstructed from Kaplan–Meier curves by application of the IPDfromKM method. These reconstructed datasets were used to perform indirect comparisons between treatments. Standard statistical testing was used, including hazard ratio and medians. A secondary analysis employed the restricted mean survival time.

**Results:**

Six trials were included in our analysis. Information on PFS from these trials was pooled according to the following treatments: nivolumab or pembrolizumab as monotherapy, or in combination with ipilimumab, and relatlimab + nivolumab. Pembrolizumab+ipilimumab showed significantly better PFS compared with the other treatments; nivolumab+ipilimumab ranked second; the other treatments showed a similar survival pattern.

**Conclusions:**

The picture of comparative effectiveness resulting from our analysis is complex. The IPDfromKM method is advantageous because it accounts for the length of follow‐up but loses the balance between treatment group and controls determined by randomization. Based on indirect comparisons, the combination of pembrolizumab+ipilimumab showed a particularly high efficacy, and so deserves further investigation. While the effect of between‐trial differences in inclusion criteria plays an important role, our results do not support the proposal of relatlimab+nivolumab as a new standard of care.

## Graphical abstract

Reconstructing individual patient data from Kaplan‐Meier curves is a new approach for studying the effect of treatments on survival endpoints. The present review describes the application of this new method in comparing immune check‐point inhibitors as first‐line in advanced melanoma.

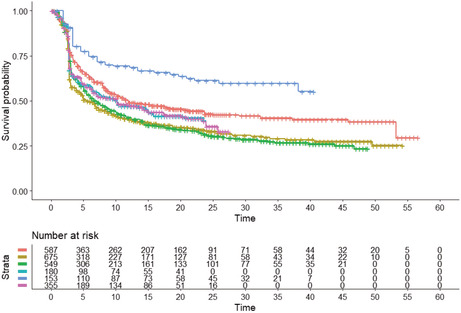

## 1 INTRODUCTION

Immune checkpoint inhibitors (ICIs) aim to disrupt the interaction between surface molecules responsible for immune exhaustion, which drives immune escape by cancer cells.^1^, ^2^, ^3^, ^4^, ^5^, ^6^ These molecules, such as programmed cell death receptor‐1 (PD‐1), cytotoxic T lymphocyte‐associated molecule‐4 (CTLA‐4) or lymphocyte‐activation gene 3 (LAG‐3), are expressed by tumor‐infiltrating T lymphocytes and, upon interaction with cognate ligands expressed by tumor cells and microenvironment, restrain immune rejection of cancer.

Advanced melanoma is the first and most meaningful test bench of immunotherapy. Anti‐PD‐1/CTLA‐4 ICI are highly effective in promoting progression free survival (PFS) and overall survival (OS) of melanoma patients and ICI have rapidly become the golden standard of care.^1^, ^2^, ^3^

While initially used as monotherapy, there was rapidly growing evidence that combination therapy was more effective. However, this success often matched with severe adverse events, which stimulate the medical research to find more appropriate dosing regimens for combination therapy.^3^

Initial trials are now mature to demonstrate that a significant proportion of patients is still alive at long follow‐up timepoints, even when treatment was discontinued. This shapes the Kaplan Meier curve with a long, elevated tail, which is not fully taken into account by traditional statistical methods.

In this scenario, it is essential to have head‐to‐head comparisons between the different ICI‐based therapeutic regimens to guide informed clinical decision and price negotiation by regulatory authorities. As this is lacking, we exploited a computerized web‐based procedure, called IPDfromKM method, to carry out the desired indirect comparisons.

The IPDfromKM method was published in June 2021 by three researchers of Department of Biostatistics of University of Texas.^7^ This method evaluates the Kaplan–Meier survival graphs using an automated analysis that reconstructs individual patient data from the time‐to‐event curve and from basic information published in the original articles (namely: number of patients and number of events). After these reconstructed databases are generated, the treatments under examination can be compared through an indirect design, by application of commonly used statistical tests. In the IPDfromKM method, on the one hand, patients studied in different trials are pooled into a single analysis and subjected to indirect comparisons, but on the other, this analysis accounts for the length of follow‐up in individual studies.

Standard survival meta‐analysis in which hazard ratios (HRs) are pooled into a single analysis is unable to account for follow‐up length, which in fact is not taken into account. The approximation is generally made that event risks are constant over time, but this assumption is known to be untrue in many survival datasets and particularly in the case of ICIs in melanoma. On the other hand, the IPDfromKM method has a disadvantage in that, while randomization associates each treatment group of each trial with its own control group, this association goes lost in the analyses based on the IPDfromKM method.

A number of studies have already employed the IPDfromKM method.^8^, ^9^, ^10^, ^11^, ^12^, ^13^, ^14^, ^15^ Apart from the complex fitting algorithm of this technique, one advantage of this method is that the indirect comparisons between survival curves are conceptually simple.

In the present work, we applied the IPDfromKM approach to study the effectiveness, in terms of progression‐free survival (PFS), of the main first‐line treatments proposed thus far for patients with advanced melanoma.

## 2 MATERIALS AND METHODS

After a standard literature search, individual patient data were reconstructed; a statistical analysis was then performed based on reconstructed patient‐level data.

### 2.1 Literature search

We carried out a literature search to identify the randomized controlled trials (RCTs) eligible for the analysis. This search was conducted in PubMed (last query on March 12, 2022) and covered the period from January 2010 to present date. A multiple search term [namely: “(ipilimumab OR nivolumab OR pembrolizumab OR relatlimab) AND melanoma[titl]”] combined with the filter “clinical trial” was used. The pathway of trial selection was handled according to the Preferred Reporting Items for Systemic Review and Meta‐Analyses (PRISMA) approach.^16^ We also searched in the Cochrane Library for any systematic review on the subject, the ClinicalTrials.gov database, and the websites of U.S. Food and Drug Administration (FDA) and European Medicines Agency (EMA). The above keywords were employed also for these additional searches.

The inclusion criteria of our analysis were: (a) previously untreated patients with advanced or metastatic melanoma; (b) phase II or phase III trial; (c) treatment arm receiving ipilimumab or nivolumab or pembrolizumab or relatlimab or any combination of these agents; (d) time‐to‐progression endpoint (i.e., PFS) reported as a Kaplan–Meier curve. For each trial, we extracted the basic information needed for our analysis (i.e., information on disease condition at baseline, number of enrolled patients and number of patients experiencing progression). Our literature search was handled according to the PRISMA flowchart.^16^

### 2.2 Reconstruction of individual patient data and

In analyzing each treatment arm of each trial, firstly we reconstructed individual‐patient data from the Kaplan–Meier curve. For this purpose, each of the 10 Kaplan–Meier curves was firstly digitized using Webplotdigitizer (version 4.5 online; url https://apps.automeris.io/wpd/); then, the progression‐free Kaplan–Meier curves, in digitized form, were input into the “Reconstruct individual patient data” subroutine of the IPDfromKM software^7^ (version: 1.2.2.0 online; last update: 1 April 2021); the total number of patients and the total number of events were input as well. Application of this subroutine generated as many sets of individual patient data as the number of enrolled patient groups. Both Webplotdigitizer and the IPDfromKM method are freely available on the Internet.

### 2.3 Statistical analysis of reconstructed patient‐level data

We carried out a standard statistical analysis for time‐to‐event endpoint (Cox statistics; package “survival”; R‐platform, 2020, https://www.R‐project.org/). The hazard ratio (HR) with 95% confidence interval [CI] was the parameter employed in these analyses. Medians of progression‐free time (with 95% CI) were also determined. As regards indirect comparisons, we planned to compare reconstructed curves among relatlimab, nivolumab monotherapy, pembrolizumab monotherapy, and combinations of two ICIs (“First analysis”). Furthermore, because the most recent trial in this area has been based on the assumption that nivolumab monotherapy can be considered the current standard of care^17^ (SOC), we compared PFS between patients treated with nivolumab monotherapy versus those treated with the combination of nivolumab plus ipilimumab (“Second analysis”).

Survival datasets including cancer patients treated with ICIs may violate the proportional risk assumption on which the Cox analysis is based; in fact, the presence of cured patients can determine a survival plateau which represents a bias in the context of the Cox statistics. For this reason, a secondary analysis was carried out in which the indirect comparisons of our first analysis were re‐assessed using the restricted mean survival time (RMST) instead of the HR estimated by Cox modeling. The RMST is not influenced by the presence of cured patients.^18^

## 3 RESULTS

### 3.1 Included clinical trials

**FIGURE 1:**
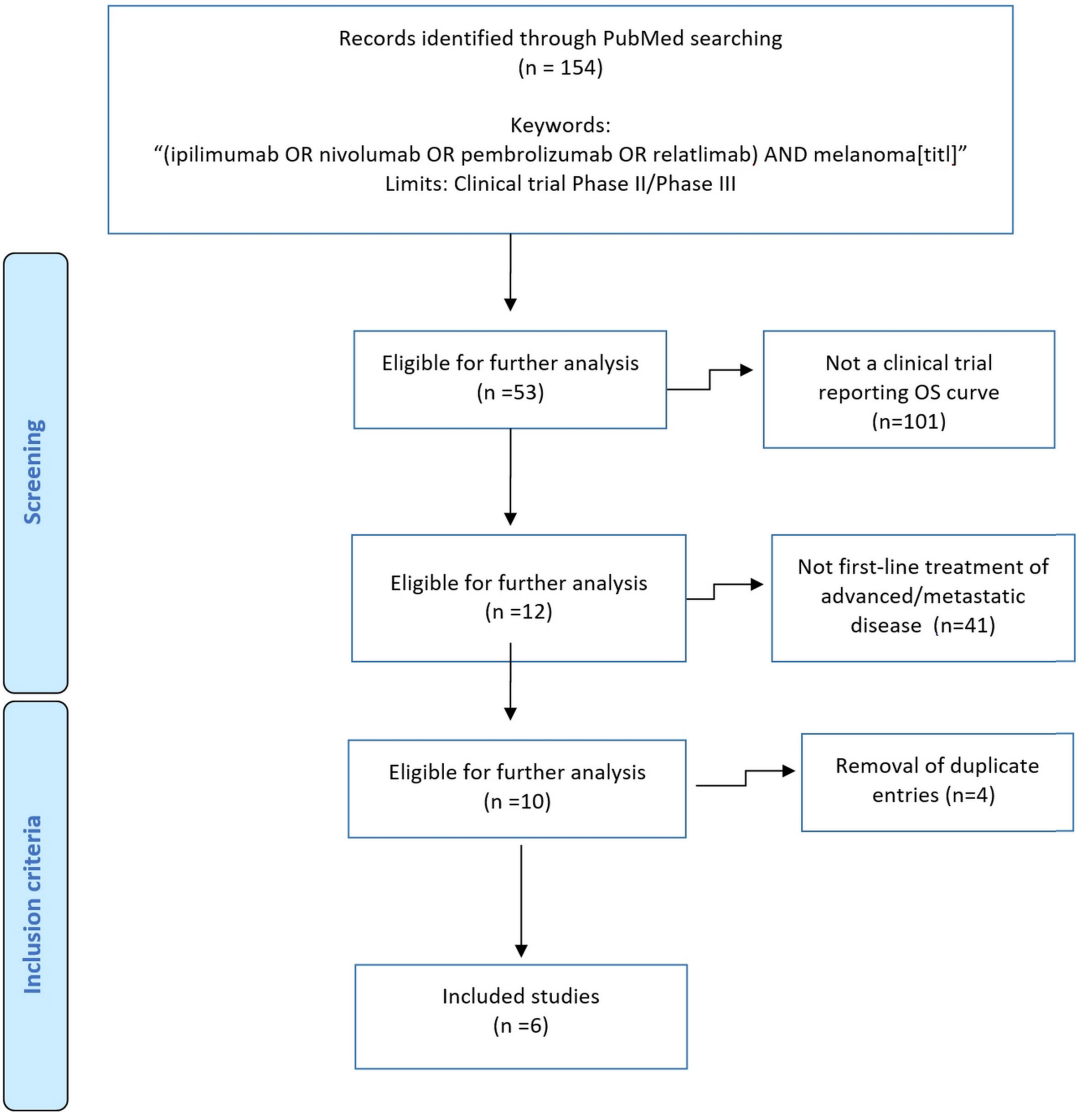
PRISMA flow diagram of our literature search.

Our literature search extracted 154 eligible papers. Duplicate entries of the same trial were managed by retaining the most recent publication reporting the longest follow‐up. Finally, six trials met the criteria for inclusion in our analysis.^17^, ^19^, ^20^, ^21^, ^22^, ^23^ Figure 1 shows the PRISMA flowchart of this literature search. Table 1 reports some basic information about these six trials.

1. RELATIVITY‐047 was a phase 2–3, double‐blind trial, where patients were randomly assigned in a 1:1 ratio to receive an anti‐LAG‐3 antibody relatlimab (160 mg) and 480 mg of nivolumab in a fixed‐dose combination or 480 mg of nivolumab.^17^
2. *Checkmate‐*067 was a phase 3, randomized, double‐blind study of nivolumab monotherapy (3 mg/kg) or nivolumab (1 mg/kg) combined with ipilimumab (3 mg/kg) every 3 weeks for four doses, followed by nivolumab (3 mg per kilogram every 2 weeks) versus ipilimumab monotherapy (3 mg/kg) in subjects with previously untreated unresectable or metastatic melanoma.^19^
3. *Checkmate‐*511 study was a phase IIIb/IV, randomized, double‐blind study where the approved regimen of nivolumab 1 mg/kg + ipilimumab 3 mg/kg once every three weeks for four doses was compared with the same schedule but different dosage, namely, nivolumab 3 mg/kg + ipilimumab 3 mg/kg. Induction phase with combination therapy was followed by maintenance with nivolumab 480 mg once every 4 weeks until disease progression or unacceptable toxicity.^20^
4. *Checkmate‐*069 Trial was a double‐blind, randomized, controlled, phase 2 trial (*Checkmate‐*069) where patients were assigned to receive nivolumab 1 mg/kg plus ipilimumab 3 mg/kg or ipilimumab 3 mg/kg plus placebo, every 3 weeks for four doses. Subsequently, patients assigned to nivolumab plus ipilimumab received nivolumab 3 mg/kg every 2 weeks until disease progression or unacceptable toxicity, whereas patients allocated to ipilimumab alone received placebo every 2 weeks during this phase.^21^
5. *Keynote*‐006 was an open‐label, multicenter, randomized, controlled, phase 3 study in which patients were randomly assigned (1:1:1) to pembrolizumab 10 mg/kg every 2 weeks or every 3 weeks or four doses of ipilimumab 3 mg/kg every 3 weeks. Pembrolizumab treatment continued for up to 24 months.^22^
6. *Keynote*‐029 Part 1B was an expansion cohort of the open‐label, phase Ib portion of *Keynote*‐029; patients received pembrolizumab 2 mg/kg (amended to 200 mg) every 3 weeks plus ipilimumab 1 mg/kg every 3 weeks (four cycles), then pembrolizumab alone for up to 2 years.^23^

**TABLE 1:** Basic information about included trials.^a^

Trial	Reference	Treatments under comparison	Treatment group (events/patients)	Controls (events/patients)
RELATIVITY‐047 (two‐arm)	Tawbi et al.^17^	relatlimab (160 mg) plus nivolumab (480 mg) nivolumab (480 mg)	180/355	211/359
*Checkmate‐*067 Trial (three‐arm)^b^	Wolchok et al.^19^	nivolumab (1 mg/kg) plus ipilimumab (3 mg/kg) every 3 weeks for four doses, followed by nivolumab (3 mg per kilogram every 2 weeks) nivolumab monotherapy (3 mg/kg)	182/314	201/316
*Checkmate‐*511 Trial (two‐arm)	Lebbè et al.^20^	nivolumab 1 mg/kg plus ipilimumab 3 mg/kg once every three weeks for four doses nivolumab 3 mg/kg plus ipilimumab 1 mg/kg. Notes: Induction phase with combination therapy was followed by maintenance with nivolumab 480 mg once every 4 weeks until disease progression or unacceptable toxicity	124/178	131/180
*Checkmate‐*069 Trial (two‐arm)^c^	Hodi et al.^21^	nivolumab 1 mg/kg plus ipilimumab 3 mg/kg every 3 weeks for four doses. Notes: patients assigned to nivolumab plus ipilimumab received nivolumab 3 mg/kg every 2 weeks until disease progression or unacceptable toxicity	95	NA^c^
*Keynote*‐006 (three‐arm)^d^	Robert et al.^22^	pembrolizumab 10 mg/kg every 2 weeks pembrolizumab 10 mg/kg every 3 weeks Notes: Pembrolizumab treatment continued for up to 24 months.	234/368	138/181
*Keynote*‐029 (single‐arm)	Carlino et al.^23^	pembrolizumab 2 mg/kg (amended to 200 mg) every 3 weeks plus ipilimumab 1 mg/kg every 3 weeks (four cycles), followed by pembrolizumab alone for up to 2 years.	61/153	NA

^a^ All values of event number were explicitly reported in the original trials with the exception of the two curves published by Lebbè et al. where we determined censored patients by counting vertical tick marks in the Kaplan–Meier curves; events in these two curves were then calculated as difference of total number of patients minus total number of censored cases.

^b^ *Checkmate*‐067 included an arm treated with four doses of ipilimumab 3 mg/kg every 3 weeks, which has not been included in our analysis.

^c^ *Checkmate*‐069 included an arm treated with four doses of ipilimumab 3 mg/kg every 3 weeks, which has not been included in our analysis.

^d^ *Keynote*‐006 included an arm treated with four doses of ipilimumab 3 mg/kg every 3 weeks, which has not been included in our analysis.

### 3.2 Generation of reconstructed time‐to‐event curves

#### 3.2.1 First analysis

In this analysis, nivolumab + ipilimumab was considered the standard of care (SOC) against which the other treatments were compared. While this reflects a wide recognition of this treatment as SOC, one point of controversy is that Tawbi and coworkers instead chose nivolumab monotherapy for the control group in their randomized trial.^17^ For this reason, an indirect comparison between relatlimab + nivolumab and nivolumab + ipilimumab was worthwhile and was carried out in this first analysis.

**FIGURE 2:**
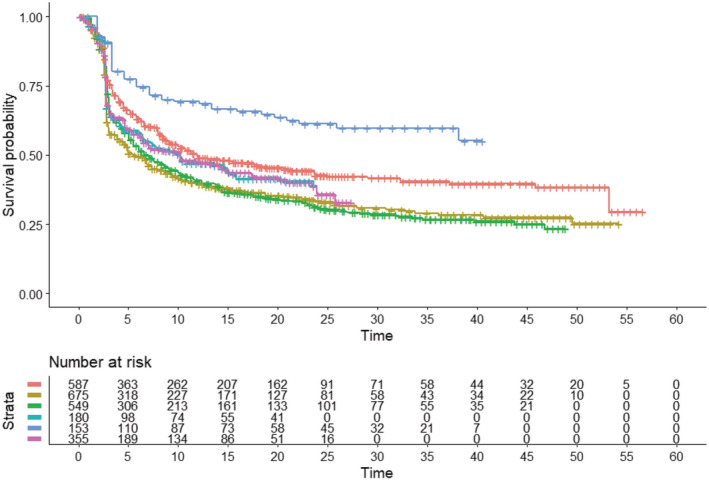
After reconstruction of individual patient data from included trials, the following Kaplan–Meier curves were generated (6 treatments): nivolumab 1 mg/kg + ipilimumab 3 mg/kg (in red); nivolumab monotherapy (in dark green); pembrolizumab monotherapy (in light green); nivolumab 3 mg/kg + ipilimumab 1 mg/kg (in light blue); pembrolizumab + ipilimumab (in dark blue); relatlimab + nivolumab (in purple). Endpoint, progression‐free survival (PFS); time in months. The number of patients for the 6 cohorts were the following: red curve (*n* = 587) from Checkmate‐067, *Checkmate*‐511, and Checkmate‐069 trials; dark green curve (*n* = 675) from RELATIVITY‐047 and *Checkmate*‐067 trials; light green curve (*n* = 549) from *Keynote*‐006 trial; light blue curve (*n* = 180) from *Checkmate*‐511 trial; dark blue curve (*n* = 153) from *Keynote*‐029 trial; purple curve (*n* = 355) from RELATIVITY‐047 trial.

For this purpose, patients receiving nivolumab + ipilimumab were pooled into a first group given ipilimumab 1 mg/kg + nivolumab 3 mg/kg (*n* = 587 from *Checkmate‐*067, *Checkmate‐*511, and *Checkmate‐*069 trials) and a second group given ipilimumab 3 mg/kg + nivolumab 1 mg/kg (*n* = 180 from *Checkmate‐*511). Also, the curves for pembrolizumab monotherapy (*n* = 368 + 181 = 549 from *Keynote*‐006 trial) and nivolumab monotherapy (*n* = 359 + 316 = 675 from RELATIVITY‐047 and *Checkmate‐*067 trials, respectively) were generated from reconstructed patient data and, finally, the reconstructed curve for the combination of pembrolizumab + ipilimumab (*n* = 153 from *Keynote*‐069 trial) was generated as well (Figure 2).

#### 3.2.2 Second analysis

The question addressed by this second analysis was whether nivolumab monotherapy can currently be considered an adequate SOC. This question is particularly relevant to appropriately interpret the results of the recent trial published by Tawbi et al.^17^ in which an innovative treatment (relatlimab plus nivolumab) was tested, and a control group treated with nivolumab monotherapy was assumed to be the SOC.

**FIGURE 3:**
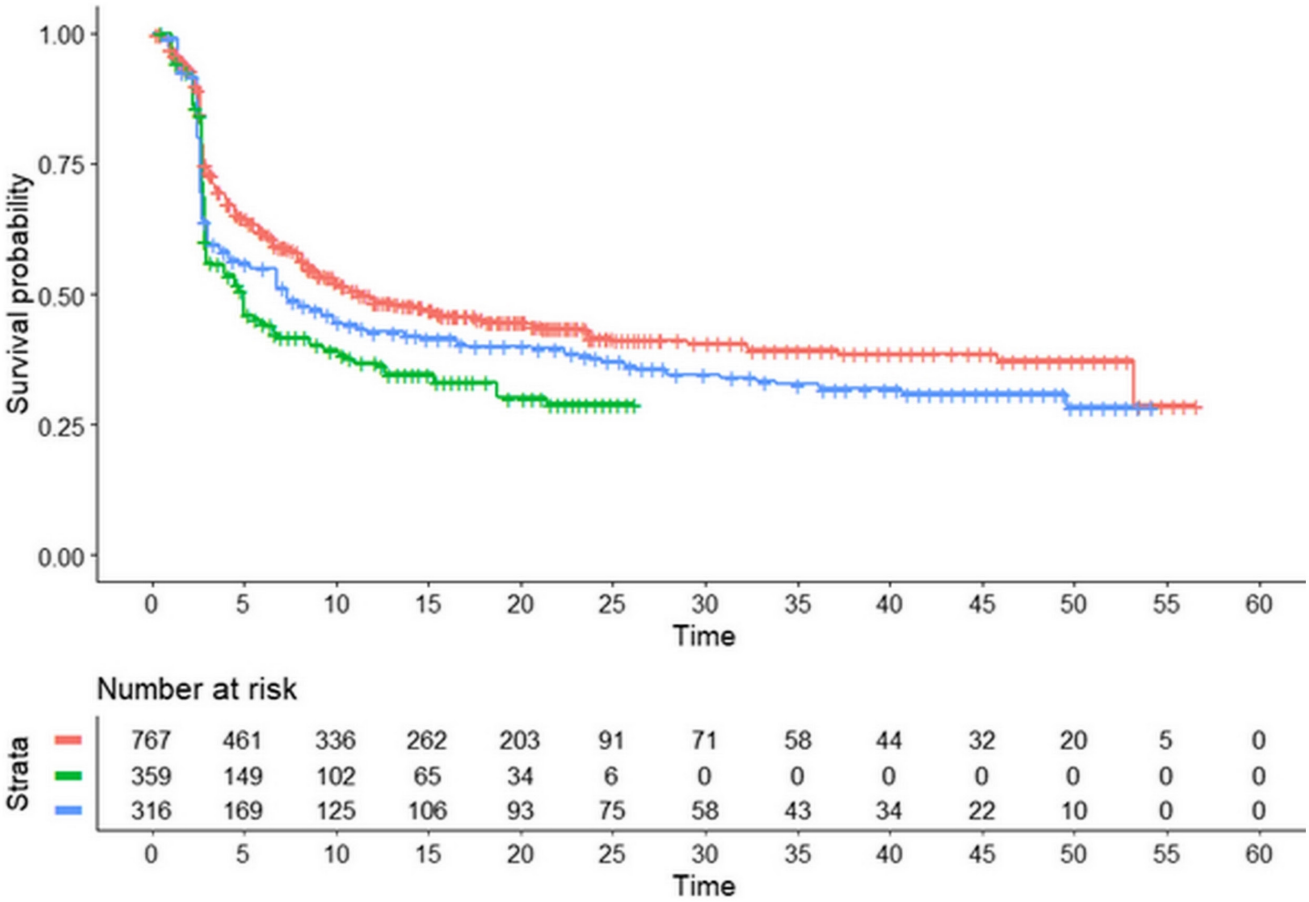
After reconstruction of individual patient data from four trials,^17^, ^19^, ^20^, ^21^ the following Kaplan–Meier curves were generated: ipilimumab+nivolumab at various dosages (*n* = 767; 4 cohorts from three trials^19^, ^20^, ^21^; in red); nivolumab (*n* = 359 from Tawbi's trial^17^; in green); nivolumab (*n* = 316 from Wolchok's trial^19^; in blue). Compared with Figure 2, in this figure the two groups treated with nivolumab + ipilimumab (with 587 and 180 patients) have been pooled together, while the group treated with nivolumab (with 675 patients) has been split into two cohorts (with 359 and 316 patients).

In our analysis based on reconstructed data (Figure 3), the survival curve for nivolumab monotherapy reported in RELATIVITY‐047 trial was compared with the curve of another trial evaluating nivolumab monotherapy (*Checkmate‐*511^20^) and with the pooled survival curve of 4 cohorts from 3 trials evaluating nivolumab + ipilimumab (*Checkmate‐*067,^19^ *Checkmate‐*511,^20^ and *Checkmate‐*069^21^). The results of these indirect comparisons (Figure 3) show that the survival pattern of Tawbi's controls^17^ is consistent with that of the other trial on nivolumab monotherapy (*Checkmate‐*067^19^), but it determines a worse PFS than that reported for nivolumab+ipilimumab in the above‐mentioned 3 trials. Hence, these indirect comparisons suggest that the control group of Tawbi et al. does not represent the best SOC from current treatments. This likely led to overestimating the efficacy of relatlimab + nivolumab. Overall, further comparative research is therefore needed to better define the place in therapy of relatlimab + nivolumab.

### 3.3 Numerical results of statistical comparisons

The numerical results of our analyses shown in Figures 2 and 3 were the following.

Regarding medians of PFS, their values (ranked in descending order) were the following:

1. Pembrolizumab + ipilimumab (*n* = 153; in dark blue): not reached (95% CI, 38.19 months to not computable);
2. Nivolumab 1 mg/kg + ipilimumab 3 mg/kg (*n* = 587; in red): 11.86 months (95% CI, 9.41 to 20.56);
3. Relatlimab + nivolumab (*n* = 355; in purple): 10.21 months (95% CI, 6.45 to 14.90);
4. Nivolumab 3 mg/kg + ipilimumab 1 mg/kg (*n* = 180; in light blue): 10.09 months (95%CI, 6.39 to 20.44);
5. Pembrolizumab monotherapy (*n* = 549; in light green): 7.03 months (95% CI, 5.54 to 8.97);
6. Nivolumab monotherapy (*n* = 675; in dark green): 5.43 months (95% CI, 4.75 to 7.33).

In our first analysis (Figure 2), using nivolumab 1 mg/kg + ipilimumab 3 mg/kg (*n* = 587) as a common comparator, the values of HR for individual treatments were the following:

1. Nivolumab monotherapy (*n* = 675): 1.42 (95% CI, 1.23 to 1.64; *p* < 0.001);
2. Pembrolizumab monotherapy (*n* = 549): 1.35 (95% CI, 1.16 to 1.57; *p* < 0.001);
3. Nivolumab 3 mg/kg + ipilimumab 1 mg/kg (*n* = 180): 1.18 (95% CI, 0.94 to 1.48; *p* = 0.15);
4. Pembrolizumab  ipilimumab (*n* = 153): 0.58 (95% CI, 0.44 to 0.78; *p* < 0.001);
5. Relatlimab + nivolumab (*n* = 355): 1.18 (95% CI, 0.99 to 1.42; *p* = 0.07).

In our second analysis (Figure 3), using nivolumab+ipilimumab at various dosages (*n* = 767) as a common comparator, the values of HR for individual treatments were the following:

1. nivolumab monotherapy (according to Tawbi et al.'s trial, *n* = 359): 1.46 (95%CI, 1.24 to 1.72; *p* < 0.001);
2. nivolumab monotherapy (according to Wolchok et al.'s trial, *n* = 316): 1.25 (95%CI, 1.06 to 1.48; *p* < 0.001).

These results indicate that: (i) monotherapies with either nivolumab or pembrolizumab fared significantly worse than the comparator (which was nivolumab 1 mg/kg + ipilimumab 3 mg/kg in our first analysis and nivolumab + ipilimumab at various dosages in our second analysis); (ii) both combinations of relatlimab + nivolumab and nivolumab 3 mg/kg + ipilimumab 1 mg/kg did not differ from the regimen chosen as comparator (which was ‐as pointed out above‐ nivolumab 1 mg/kg + ipilimumab 3 mg/kg in our first analysis and nivolumab+ipilimumab at various dosages in our second analysis); (iii) the combination of pembrolizumab + ipilimumab determined a significantly better PFS than nivolumab 1 mg/kg + ipilimumab 3 mg/kg.

Further indirect comparisons across treatments were the following:

1. The combination of relatlimab + nivolumab showed a significantly worse PFS compared with pembrolizumab + ipilimumab (HR, 2.07; 95% CI, 1.52 to 2.82; *p* < 0.001);
2. Relatlimab + nivolumab (*n* = 355) showed a numerically better PFS compared with nivolumab monotherapy (*n* = 359 + 316 = 675, i.e. the two patient groups pooled from RELATIVITY‐047 and *Checkmate*‐067 trials), but the difference did not reach statistical significance (HR, 1.20; 95% CI, 0.95 to 1.51; *p* > 0.05). Differently, the group treated with relatlimab+ nivolumab (*n* = 355), in comparison with only the nivolumab monotherapy group of RELAVITY‐47 trial (*n* = 359), showed a statistically significant difference (HR, 0.77; 95% CI, 0.63 to 0.93; *p* = 0.008). Interestingly, this result from reconstructed patient data is nearly identical to that obtained from “real” individual patients in the RELATIVITY‐047 trial (HR, 0.75; 95% CI, 0.62 to 0.92; *p* = 0.006) and published in the original study.

Finally, our secondary analyses based on the RMST provided essentially the same statistical results as those obtained using the Cox model combined with HRs (see Tables A1 and A2 in the Appendix). Hence, the presence of cured patients did not affect the statistical results of our two primary analyses obtained with the Cox model and expressed according to the HRs.

## 4 DISCUSSION

The present work can be of interest especially because an original approach (the IPDfromKM method) has been used for survival analysis. The main feature of this method is that individual patient data are reconstructed from the Kaplan–Meier curves published in the original trials. In this reconstruction, individual patients are identified along with their follow‐up length and their outcome (i.e., cases with event or right‐censored cases). Firstly, these reconstruction procedures are carried out separately for each patient cohort; then, the subsequent statistical analysis can pool pertinent patients irrespective of their origin. Indirect comparisons are finally performed by using standard statistical tests (such as log‐rank test, Cox model, hazard ratio, Kaplan–Meier graphs based on reconstructed patients, and even more specialized statistical analyses like those based on the restricted mean survival time). It should be stressed that the IPDfromKM method is specifically designed to manage time‐to‐event endpoints, which represent an area where a consensus has not been reached on how a meta‐analysis can be conducted.

One strength of the IPDfromKM method is related to the simple graphical presentation of its results. This format, based on a single multi‐treatment Kaplan–Meier graph, balances simplicity with completeness, and its strong level of synthesis is a key advantage. As regards limitations of the present work, the indirect nature of the comparisons, already discussed above, remains the most important one. Another limitation is that our analysis evaluated only PFS as primary outcome and did not study overall survival owing to the insufficient material available on this endpoint.

In the application of the IPDfromKM method, heterogeneity testing has been managed in previous studies by conducting a separate analysis in which the Kaplan–Meier curves of control groups are compared with one another to assess the extent of between‐trial differences.^10^, ^11^, ^12^, ^13^, ^14^ In the present work, this analysis has been split into a first comparison including patient groups treated with nivolumab monotherapy, and a second comparison including patient groups treated with nivolumab plus ipilimumab. This approach of heterogeneity assessment has an empiric nature given that a method to optimally manage heterogeneity within reconstructed patient databases still needs to be defined. Anyhow, as regards the issue of heterogeneity related to differences among control groups, the solution likely lies in the clinical side of the problem, and a careful examination is needed of the potential effects of different inclusion and exclusion criteria among trials.

Regarding the findings of our survival analysis, the combination of pembrolizumab + ipilimumab quite unexpectedly determined a better PFS than each of the remaining treatments. In a separate analysis where we considered pembrolizumab + ipilimumab as a common comparator, the PFS of this treatment was significantly better than that of each of the other 5 treatments (5 indirect comparisons, all with *p* < 0.001; detailed results not shown).

This excellent performance of pembrolizumab + ipilimumab in *KEYNOTE*‐029 trial was pointed out by Carlino et al. in the discussion of their study.^23^ Carlino et al.^23^ stressed that cross‐trial comparisons should be interpreted cautiously, given differences in study design and patient populations. While this point is unquestionable, Carlino et al. nevertheless offered a number of explanations for this finding. First, in *KEYNOTE*‐029 compared with *Checkmate‐*067, a lower proportion of patients had the poor prognostic feature of elevated lactate dehydrogenase levels (25% vs. 36%). On the other hand, in *KEYNOTE*‐029, 13.1% of patients had previously received therapy, most commonly BRAF and/or MEK inhibitors, a factor associated with reduced response to checkpoint inhibition, whereas in *Checkmate‐*067 all patients were treatment naive. The proportion of patients with PD‐L1–positive disease also has the potential to complicate comparisons of efficacy between studies, but (as pointed out by Carlino et al.^23^) PD‐L1 status alone is unlikely to be a strong determinant of outcome in patients treated with the combination of nivolumab and ipilimumab. Overall, the most plausible explanation for the improved PFS seen in *Keynote*‐029 may be toxicity management. Indeed, initial ipilimumab + nivolumab studies mandated cessation of both agents in response to significant toxicity. In contrast, in *Keynote*‐029 ipilimumab could be ceased until resolution of toxicity grade 1 or less, whereas pembrolizumab could be continued at the investigator's discretion. The ability to continue pembrolizumab may be responsible for the favorable duration of response seen in *Keynote*‐029 and the consequent favorable PFS. Another explanation is that the use of systemic corticosteroids to manage immune‐mediated adverse events was different in *Keynote*‐029 and *Checkmate‐*067 (69.1% in *Keynote*‐029; 83.4% in *Checkmate‐*067), but the impact of corticosteroid uses on survival outcomes of patients receiving pembrolizumab or nivolumab remains to be determined.

In conclusion, the present analysis has comparatively examined the main trials that have so far evaluated first‐line treatments for advanced melanoma. Our main result is that, in comparing the RELATIVITY‐047 trial with the other trials published over the last years, relatlimab + nivolumab cannot be considered the new SOC mainly because the control group of that trial did not receive the best treatment available.

Hence, the combination of nivolumab + ipilimumab in our view, remains the best SOC. In fact, our findings confirmed the excellent PFS observed with the combination of pembrolizumab + ipilimumab indicating that this treatment could be significantly better than the others. As Carlino et al.'s study does not have a control group, it should however be stressed that a further clinical proof is still needed before this conclusion can be accepted. Caution is also needed in consideration of our methodological approach based on indirect comparisons; in fact, indirect comparisons of reconstructed patient‐level data should only be considered hypothesis generating and should not be taken as proof of significant differences in the benefit of the various regimens.

## AUTHOR CONTRIBUTION

AM and PB designed the study, MC and DM performed the literature search and extracted information from included studies, AO and VD ran the IPDfromKM software, AM, VD, and AO wrote the manuscript in consultation with MC, PB, and DM.

**Note:** Figure 2 is designated for Graphical Table of contents in the ScholarOne system.

## FUNDING INFORMATION

None.

## CONFLICT OF INTEREST

The authors declare no conflict of interest.

## ETHICS STATEMENT

The current study is exempt from ethical approval because no human participants were enrolled for the purposes of the present work.

## Data Availability

The data used for the application of the IPDfromKM method are available from the authors upon request.

## APPENDIX A

This Appendix includes Tables A1 and  A2.

**TABLE A1:** Indirect comparisons of our first analysis reassessed using RMST instead of HR

Comparison		Milestone (months)	RMST (months)		Difference (months)	Statistical significance	
First comparator	Second comparator		First comparator	Second comparator		According to RMST	According to HR^a^
nivolumab 1 mg/kg + ipilimumab 3 mg/kg	nivolumab monotherapy	54.23	25.834, *n* = 587	19.999, *n* = 675	5.836 (95%CI, 3.017 to 8.654)	*p* < 0.001	*p* < 0.001
	pembrolizumab monotherapy	48.87	23.866, *n* = 587	18.311, *n* = 549	5.555 (95%CI, 3.029 to 8.081)	*p* < 0.001	*p* < 0.001
	nivolumab 3 mg/kg + ipilimumab 1 mg/kg	23.57	13.615, *n* = 587	12.635, *n* = 180	0.980 (95%CI, −0.689 to 2.648)	*p* = 0.25	*p* = 0.15
	pembrolizumab + ipilimumab	40.51	20.589, *n* = 587	27.131, *n* = 153	−6.542 (95%CI, −9.740 to −3.343)	*p* < 0.001	*p* < 0.001
	relatlimab + nivolumab	27.34	15.215, *n* = 587	13.982, *n* = 355	1.232 (95%CI, −0.343 to 2.808)	*p* = 0.125	*p* = 0.07

*Note*: Abbreviations: CI, confidence interval; HR, hazard ratio; RMST, restricted mean survival time.

^a^ These *p*‐values are those estimated in our first analysis.

**TABLE A2:** Indirect comparisons of our second analysis reassessed using RMST instead of HR

Comparison		Milestone (months)	RMST (months)		Difference (months)	Statistical significance	
First comparator	Second comparator		First comparator	Second comparator		According to RMST	According to HR^a^
nivolumab + ipilimumab (various dosages)	nivolumab monotherapy (Tawbi et al.)	26.18	14.466, *n* = 767	11.181, *n* = 359	3.285 (95%CI, 1.890 to 4.680)	*p* < 0.001	*p* < 0.001
	nivolumab monotherapy (Wolchokk et al.)	54.23	25.276, *n* = 767	21.887, *n* = 316	3.389 ‐(95%CI, 0.151 to 6.627)	*p* = 0.04	*p* < 0.01

*Note*:Abbreviations: CI, confidence interval; HR, hazard ratio; RMST, restricted mean survival time.

^a^ These *p*‐values are those estimated in our second analysis.
